# Design and Validation of a Scalable, Reconfigurable and Low-Cost Structural Health Monitoring System

**DOI:** 10.3390/s21020648

**Published:** 2021-01-19

**Authors:** Juan J. Villacorta, Lara del-Val, Roberto D. Martínez, José-Antonio Balmori, Álvaro Magdaleno, Gamaliel López, Alberto Izquierdo, Antolín Lorenzana, Luis-Alfonso Basterra

**Affiliations:** 1 Department of Signal Theory and Communications and Telematic Engineering, University of Valladolid, Paseo de Belén, 15, 47011 Valladolid, Spain; alberto.izquierdo@tel.uva.es; 2 School of Industrial Engineering, University of Valladolid, Paseo del Cauce 59, 47011 Valladolid, Spain; alvaro.magdaleno@uva.es (Á.M.); ali@eii.uva.es (A.L.); 3 Research group of Timber Structures and Wood Technology, University of Valladolid, Av. de Salamanca, 18, 47014 Valladolid, Spain; robertomartinez@arq.uva.es (R.D.M.); balmori@arq.uva.es (J.-A.B.); gama@arq.uva.es (G.L.); basterra@arq.uva.es (L.-A.B.)

**Keywords:** low-cost SHM, MEMS accelerometers, myRIO platform, non-destructive testing

## Abstract

This paper presents the design, development and testing of a low-cost Structural Health Monitoring (SHM) system based on MEMS (Micro Electro-Mechanical Systems) triaxial accelerometers. A new control system composed by a myRIO platform, managed by specific LabVIEW software, has been developed. The LabVIEW software also computes the frequency response functions for the subsequent modal analysis. The proposed SHM system was validated by comparing the data measured by this set-up with a conventional SHM system based on piezoelectric accelerometers. After carrying out some validation tests, a high correlation can be appreciated in the behavior of both systems, being possible to conclude that the proposed system is sufficiently accurate and sensitive for operative purposes, apart from being significantly more affordable than the traditional one.

## 1. Introduction

Today, both the construction of new buildings and the conservation of cultural heritage and the rehabilitation of buildings, due to the boom in sustainability, are taking on great importance. The diagnosis, restoration and conservation of architectural heritage structures require a thorough knowledge of the characteristics of the structure and the materials they are made of, as well as their response throughout their span life. The number of old exiting buildings and civil structures (i.e., resident buildings, hospitals, bridges, …) that need an adequate control and maintenance to guarantee their structural operation and safety is currently huge. Furthermore, Spain is the third country with the largest number of historic buildings (after China and Italy) [1], whose adequate maintenance is essential, complex and unaffordable considering the cost and abilities of the current SHM (Structural Health Monitoring) devices. Only with a new generation of them, feasible structural assessment can be carried out, not only for existing buildings and structures but also for new ones.

A continuous monitoring of the structural integrity could help to detect premature aging or damaging and guide an adequate maintenance plan considering the limited funding. These monitoring techniques can include a data logger device and several sensor units, and, as in the proposed case, some processing capacity prior to the data transfer process [2,3,4].

Vibration testing is an accurate structural assessment in which the use of accelerometers (among other types of sensors) allows the measurement of the structural response and detecting changes in the modal parameters (natural frequencies, damping ratios and mode shapes) [5], giving clues about the existence of some type of structural damage [6,7]. Several examples of vibration monitoring systems for SHM in industrial machines, wind turbines, or typical civil structures such as bridges can be found in [8,9,10,11]. Nevertheless, on building structures, this technique has mainly been used as early warning systems (EWS) in seismic areas [12], and more limitedly as a non-destructive technique (NDT) on historic timber structures [13].

There are different accelerometer types and the most widely used have traditionally been the piezoelectric crystal ones, which are highly accurate and sensitive. However, these accelerometers are expensive, potentially reducing their availability for tests with large set-ups. Besides, their high economic value also limits the possibility of using them just for a short test period, and they can rarely be used for continuous real-time measurements [14]. In the last decade, new digital sensors based on the Micro Electro-Mechanical Systems (MEMS) technology have been applied to structural health monitoring with promising results [15,16,17]. These MEMS digital accelerometers provide similar measurement to traditional devices, but at a much lower price, reduced size and easy performance [18,19,20,21,22,23]. Recently, several studies have compared low-cost MEMS sensors with traditional piezo-electric accelerometers on SHM monitoring tests on real structures. These investigations have shown the technical advantages and limitations of this new digital technology [24,25,26,27,28,29]. The MEMS technology has grown quickly in the last few years, new accelerometers being developed with improved properties, accuracy and sensitivity, but keeping their small size, wide variety of available models, and very low cost. For that reasons, MEMS accelerometers are suitable for structural monitoring, and many researchers have used this technology to develop new SHM systems [23,24,25,30,31,32,33].

However, the SHM systems must comprise not only sensors but also a suitable data logger for acquiring data from them and processing the registered signals. Such an integrated systems remain a challenge, and, despite their promising features, there are still very few commercially available systems at a great cost [34,35]. To overcome this, the use of digital platforms integrating microprocessors and field-programmable gate arrays (FPGA) are proposed. Synchronous acquisition is attended by the FPGA while the processor operates with the previously registered signals.

Structural health analysis and diagnosis techniques, based on forced dynamic response or on environmental or operational modal analysis, are successfully used to assess both new buildings and historic buildings. The idea behind this technology is based on the fact that the modal parameters (natural frequencies, vibrating mode shapes and modal damping ratios) depend, among other factors, on the physical properties of the material (mass, damping and stiffness), and new accelerometers are the ideal sensors for their analysis. In recent years, the application of MEMS technologies to accelerometers has enabled their development towards very low energy consumption, low noise and high sensitivity, i.e., high-quality accelerometers, at very affordable prices. The use of wireless networks to monitor buildings, together with the use of MEMS accelerometers, makes it possible to reduce costs and mitigate the inconveniences associated with monitoring: energy consumption and autonomy, wiring, installation time, or even mitigation of its visual and physical impact (an important issue in heritage conservation).

In addition to these solutions based on MEMS sensors, in the SHM world have appeared networks with wireless sensors that are based on the paradigm Wireless Sensors Networks (WSN) [36]. These wireless architectures send data of reduced size at specific time intervals (synchronous mode) or asynchronously to specific events. These systems are very versatile and require a synchronization system for the time base is common to all sensors. Together with this fact, recently the Internet of Things (IoT) paradigm has burst, making possible the combination of sensors and low-cost acquisition systems, which can use wired and wireless networks [37], and this paradigm has also reached the SHM [38].

The architecture proposed in this paper is framed in the field of modal analysis using digital MEMS accelerometers to obtain the FRF functions and the subsequent modal analysis. Capture times of the order of tens of seconds are required, synchronously sampling the sensors at rates of up to 4000 samples/second so that the phase relationships are preserved. These restrictions require: (i) that the sensors are wired and therefore that the WSN paradigm is not applicable and (ii) that the acquisition system is based on an FPGA that ensures that the sampling is synchronous, thus discarding microcontroller-based architectures (Arduino, Raspberry pi, etc.), typical of the IoT paradigm.

The main objective of this research is to develop a low-cost SHM system based on the use of wireless networks to monitor buildings, together with the use of MEMS accelerometers. A scalable, modular and reconfigurable system architecture, which includes modules of acquisition, processing and analysis for a SHM system, has been developed. This system can record multiple channels at high acquisition rates, like some other expensive commercial datalogger systems, and then sequentially process and evaluate the recorded information, which no other commercial systems do jointly; that is, these abilities are not included in standard commercial systems. In order to validate the developed monitoring system, a campaign test was carried out on a timber structure simultaneously instrumented by using the proposed system and a standard commercial system.

This paper is structured as follows: firstly, in Section 2, the characteristics of the MEMS monitoring system are enunciated together with the system requirements and the system set-up where the sensors, the acquisition system, the processing system and its architecture are described. In Section 3, the validation test and its results are described. In Section 4, the results of both systems are compared not only on the basis of the registered time signals but also in terms of the computed Frequency Response Functions (FRFs). Finally, the main conclusions are drawn and presented in Section 5.

## 2. Monitoring System Design

### 2.1. Requirements

The main objective of the designed system is to allow the monitoring of building structures using digital MEMS accelerometers. To achieve this objective, a set of requirements have been defined that must be satisfied by the system.

The designed SHM system must be able to be used in buildings and structures with very different characteristics in both their dimensions and modal properties. Therefore, one of the main requirements of the system is that it must be scalable, allowing the number of used accelerometers to be varied, and reconfigurable, so that the location of the sensors could be changed to adapt the set-up to the structure to be measured. Finally, the system must be distributed, consisting of a set of autonomous modules connected wirelessly. Each of the modules must be able to acquire and process data from a set of sensors by exchanging synchronization information with the other modules and with the control module, so as the quality of the final properties that can help to decide about the structural integrity, computed from the registered signals, is not compromised.

Other system requirements are:

- Ability to acquire a potentially high number of digital MEMS accelerometers.
- Ability to generate proper input signals to command the excitation devices, for example, an inertial shaker with different patterns: noise, tones, frequency sweeps, etc.
- Possibility of acquiring and integrating information from other sensors (load cells, temperature, humidity, etc.), both analogue and digital.
- Possibility of autonomous operation with recording in a cloud database.
- Low cost, by selecting components that allow to reduce the investment and operational cost in comparison with standard commercial systems.

### 2.2. System Arquitecture

**Figure 1:**
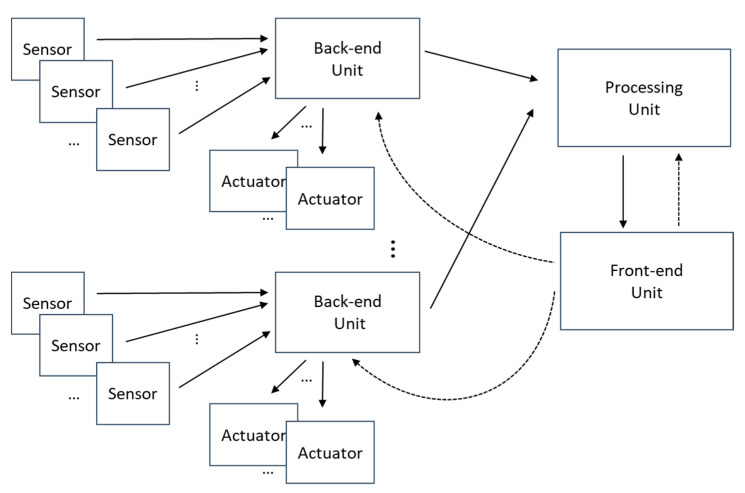
Proposed system architecture.

To achieve the proposed requirements, the system architecture that has been selected is shown in Figure 1. It can be observed that the system is formed by the sensors, the adaptation units, called the back-end units (BE-U), the processing unit (P-U) and the control unit, called the Front-end unit (FE-U).

The system’s sensors, mainly accelerometers, are divided into groups that are managed by a set of BE-Us, which are responsible for configuring and reading the data provided by each sensor. The system’s actuators such as shakers or pneumatic hammers must also be associated by a back-end unit, which will be in charge of generating and transferring the actuation signals to the device.

The sensor data obtained by the back-end acquisition units are transferred to the processing unit that is responsible for pre-processing the signals in the time domain (filtering, decimation, etc.) and perform the necessary calculations to obtain the FRFs. The processing unit is also in charge of storing the processing results in a cloud-based database. Additional post-processing such as perform the modal analysis or detect alarm situations could also be included.

Finally, the front-end unit oversees the measurement and calculation process, establishing the intervals in which the measurements are made and the configuration of the rest of the elements of the system. It is also in charge of the interaction with the user, allowing the visualization of the taken measurements and the obtained results.

#### 2.2.1. Sensor Description

**Figure 2:**
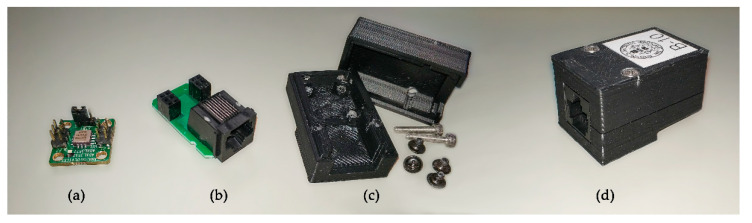
(**a**) ADXL355 accelerometer (**b**) adaptor board (**c**) 3D printed box (**d**) sensor assembled.

The selected accelerometer, shown in Figure 2a, was the ADXL355 digital MEMS accelerometer, developed by Analog Devices [39]. The ADXL355 is a low power 3-axis accelerometer with selectable measurement ranges. Particularly, it supports the ±2 g, ±4 g, and ±8 g ranges. It integrates a 20 bits sigma-delta ADC (Analog to Digital Converter) per axis that provides sensitivities of 3.9 µg/LSB (least significant bit), 7.8 µg/LSB, mV/g and 15.6 µg/LSB, respectively, with a noise density of 25 µg/√Hz, and a bandwidth up to 1500 Hz. The ADXL355 is highly integrated in a compact form factor, and its low power, with less than 200 µA current consumption, is ideal in an Internet of Things (IoT) application and other wireless product designs.

ADXL355 accelerometers measure vibrations with high resolution and very low noise to allow the early detection of structural failures, using wireless sensor networks. Their low power consumption allows for extended product use by prolonging the time between battery changes. The low noise performance of the ADXL355 series with low power consumption allows its use on low level vibration measurement applications, such as SHM, with an affordable cost.

ADXL355 accelerometers connect through a standard SPI (serial peripherical interface) that needs four digital data cables for communication, two power lines and an extra data one for synchronization. Because there must be a total of seven cables from the sensor to the acquisition device, a standard Ethernet cable with RJ45 connectors has been chosen to acquire the data from the sensors. This type of cable provides four pairs of cable lines, is available in a wide range of lengths and has very low cost. For the intended application (heritage timber building), a maximum cable length of 10 m, with cables of category 5+ FTP, has been successfully used. Different lengths have been tested, with the limit length being 10 m, since it was found that the critical point was the delays due to the length of the cable, not so much to the noise it might introduce.

A small adaptor board has been developed to join the sensor to a RJ45 connector, as can be observed in Figure 2b. The whole set has been encapsulated in a little box that has been 3D printed (Figure 2c). This box provides mechanical integrity to the sensor and provides an anchoring mechanism to the structure to be measured. Figure 2d shows the final sensor encapsulated in the box.

#### 2.2.2. Back-End Unit

A FPGA is the most suitable hardware to implement the back-end unit due to its flexibility in the control of the inputs and outputs and its high capacity to parallelize. The disadvantage of this approach is the complexity and high development time. To solve this handicap a myRIO platform [40] has been selected as the base for the adapter unit. This platform is a real-time embedded evaluation board developed by National Instruments which incorporates a FPGA that can be programmed using the LabVIEW graphical programing language.

This platform belongs to the Reconfigurable Input-Output (RIO) family of devices from National Instruments that is oriented to sensors with nonstandard acquisition procedures, allowing low-level programming of the acquisition routines. Specifically, the myRIO platform is an embedded hardware based on a Xilinx Zynq 7010 chip, which incorporates a FPGA and a dual-core ARM^®^ (Advanced RISC Machine) Cortex™-A9 processor. The FPGA has 40 lines of digital input/output that are used as the connection interface with the ADXL355 sensors, 2 AD inputs and 2 DA outputs. The ARM processor is equipped with 256 MB of DDR3 RAM (Double Data Rate 3 Random-Access Memory), 512 MB of built-in storage space, USB Host port, and Wi-Fi interface. All this hardware is enclosed in a small box (136 mm × 89 mm × 25 mm).

**Figure 3:**
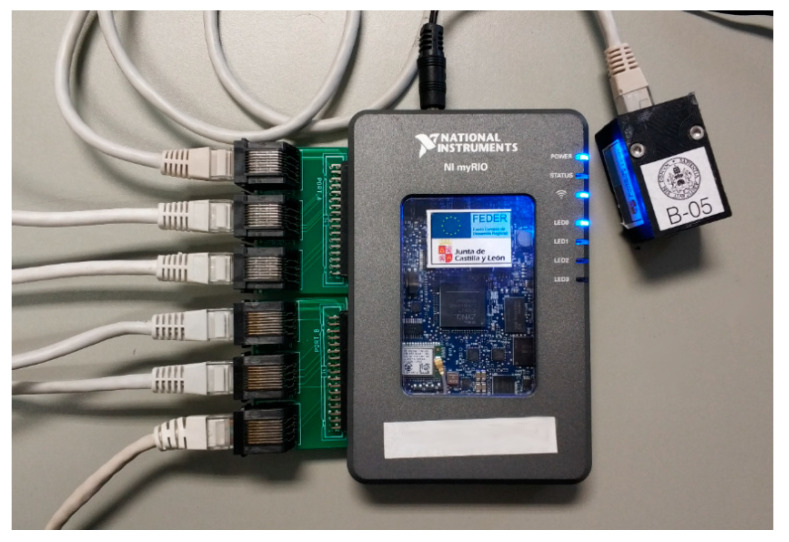
MyRIO device with two adapter boards and an accelerometer.

Both MXP ports with 16 I/O lines of the myRIO have been used to connect the ADXL355 sensors. As each sensor needs five I/O lines, up to 6 sensors can be attached to a myRIO without multiplexing I/O lines. An MXP-to-RJ45 adaptor board with 3 RJ45 connectors has been developed to allow the connection of ADXL355 sensors to the two MXP ports of the myRIO as can be shown in Figure 3.

As far as, several of the I/O lines of the SPI interface used on the ADXL355 could be shared between multiple sensors, so that, up to 16 sensors could be connected to a single myRIO if needed.

The two analog input ports of the myRIO device have been used to acquire analog data from other devices, like analog accelerometers or a load cell, synchronously with the digital data from the ADXL355 sensors. On the other hand, the analog output ports have been used to generate excitation signals used as the source to shakers or other type of modal exciters.

#### 2.2.3. Processing and Front-End Units

**Figure 4:**
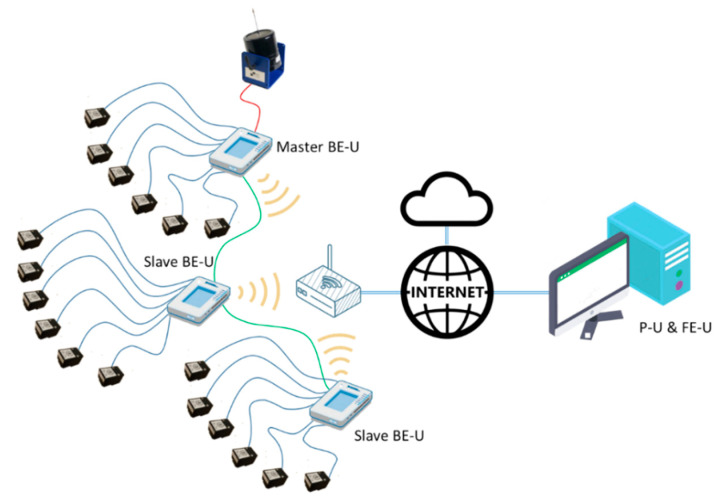
Distributed system configuration.

Different hardware could be used to implement both the processing and the front-end units. For example, Figure 4 shows a configuration were the FE-U and P-U are implemented in a PC while several myRIO devices with up to six sensors each one works as back-end units.

In this configuration, the PC performs two main functions:

- As a front-end unit, the PC manages all the myRIO platforms connected to the system using a Wi-Fi interface. The PC sends the configuration of the six accelerometer sensors attached to each myRIO device, controls when the acquisition starts and when it stops, and receives the acquired data from the accelerometers for further processing. In addition, the PC shows a user interface that allows changing the system parameters and visualizing the results of the modal analysis of the structure.
- As a processing unit, the PC could execute additional algorithms to perform the modal analysis, evaluate structural changes or generate early warning signals, among others.

**Figure 5:**
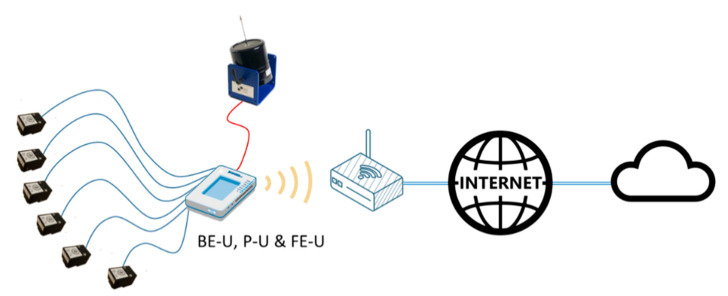
Stand-alone system configuration.

On the other hand, the functions of the myRIO devices as BE-Us are:

- Each myRIO device carries out the synchronous acquisition of the data from the attached ADXL355 sensors and the analog inputs and sends them to the PC.
- One of the myRIO can generate several types of signals in order to be used as excitation signal: a single tone of a fixed frequency, white noise within a limited frequency band or a tone sweep between two frequencies.
- If several myRIO platforms are simultaneously incorporated to the system, a synchronization mechanism must be used to ensure that the data from all the accelerometers is acquired at the same time. One of them must be the master, in order to generate a synchronize signal that is used by the myRIO slaves to start the acquisition synchronously.
- On the other hand, the ARM processor included in the myRIO platform can also be used to implement PU and FE-U at the same time as the BE-U, defining a stand-alone system as shown in Figure 5.

In this configuration, a single myRIO works as an autonomous system that performs measurements, calculates modal analysis and stores the results on the cloud. A web-based user interface can be used to interact with the stand-alone system configuring it and getting the results.

Thanks to the use of the LabVIEW graphical programming environment to implement the system, the same algorithms can be executed in different processors like a PC or an ARM embedded platform with minor or no changes in the software source code.

## 3. System Validation

In order to validate the measurements and processing algorithms of the proposed system a set of experiments have been performed. A well-probed commercial data logger has been chosen as the reference system and high-end piezo-electric accelerometers have been placed side-by-side with the digital MEMS accelerometers on a test structure in order to perform some comparison measurements.

### 3.1. Reference System

The Dewesoft platform has been selected to be used as the reference system, which is composed of a DS-SIRIUS DAQ [41] device and 6x KS76C.100 [42] accelerometers along with the DewesoftX data acquisition software.

DS-SIRIUS is a dual-core 24-bit data logger with an anti-aliasing filter on each channel with up to 200 kS/s sampling rate per channel and 160 dB of dynamic range in the time and frequency domains. The model used here (DS-SIRIUS-8xACC-8xAO) manages 8 input and 8 output channels. It is intended for IEPE sensors and supplies a configurable voltage (up to ±10 V) and current (between 4 and 8 mA). The power consumption per channel is 1 W.

The Integrated Electronics Piezo-Electric (IEPE) accelerometers KS76C.100 are intended for standard applications in laboratory and industry, for vibrations between 0.5 and 70 kHz, and require a current supply ranging between 2 and 20 mA. Its acceleration range is ±60 g with a sensitivity of 100 ± 5 mV/g and 3 µg/√Hz noise density value.

Table 1 compares the more relevant characteristics of the proposed system versus the system used as reference:

### 3.2. Measurement Layout

To carry out the tests, a sawn timber beam of Scots pine (*Pinus sylvestris* L.) with a nominal section of 90 mm × 140 mm and a length of 5000 mm, harvested from a natural forest in Cabrejas del Pinar (Soria, Spain), and bought in the sawmill of “Maderas PinoSoria”, is used. The specimen was conditioned in a climatic chamber at 20 ± 2 °C and 65 ± 5% relative humidity (approx. 12% equilibrium moisture content). The piece was previously tested in the laboratory to obtain its mechanical and elastic properties. A resistant class of C27, according to standard EN 338:2016, and a modulus of elasticity of 11,901 MPa, according to standard EN 408:2011, were estimated.

**Figure 6:**
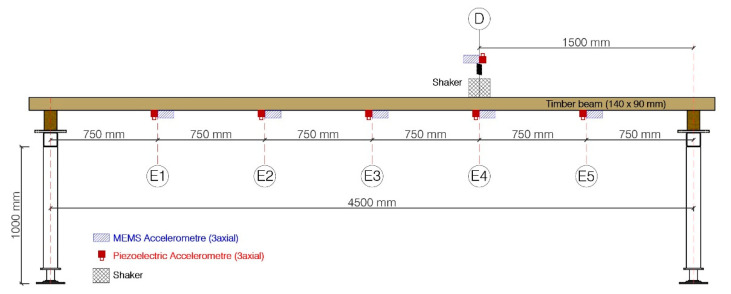
Elevation view of the measurement layout for the validation tests.

**Figure 7:**
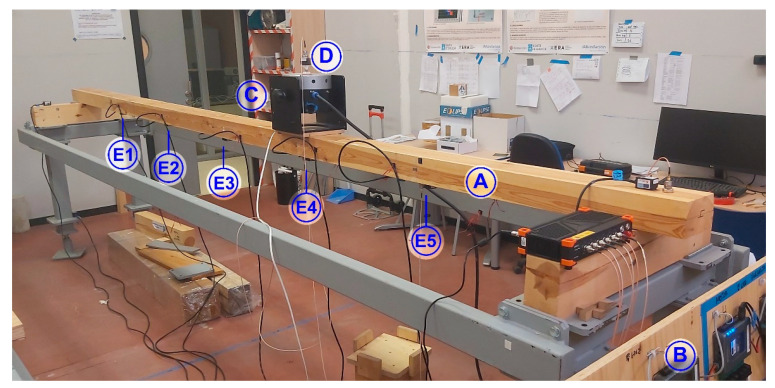
Measurement layout.

The timber beam is placed on a wooden support which is, in turn, placed on a steel frame. It lies in a horizontal position (140 mm) with a separation between supports of 4500 mm, as can be seen on Figure 6. It is instrumented with 5 pairs of accelerometers evenly distributed on the timber beam (750 mm of separation), at the positions marked as E1 to E5 on Figure 6. Each pair of accelerometers is formed by a piezoelectric accelerometer (KS76C.100) and a MEMS accelerometer (ADXL355). The beam is excited with an electromechanical shaker placed in the vicinity of the fourth pair of accelerometers, at 1500 mm from the right support, oriented in the vertical direction as can be observed in Figure 6. Additionally, another pair of accelerometers is placed on the moving mass of the shaker (0.23 kg), labeled as D, to record the input force (calculated as the measured acceleration times the value of the moving mass), as it is shown in Figure 6. The laboratory measurement layout can be observed in Figure 7. In this figure, the timber beam is marked with the letter A, the myRIO device is marked with the letter B, and the shaker is marked with the letter C.

During the comparative test, the shaker was controlled by a signal generated by the proposed system. This signal consisted of a sinusoidal sweep with constant amplitude, its frequency ranging between 3 and 50 Hz and a duration of 3 min for a single sweep. Both systems performed the acquisition simultaneously for approximately 8 min, which corresponded to almost 3 cycles of excitation.

## 4. Comparative Results

To validate the accuracy of the proposed system the response of the beam has been analyzed in both time and frequency domains. First, the acquired signals from the proposed and reference systems have been compared in the time domain and then, the Frequency Response Functions calculated by each system have also been analyzed.

### 4.1. Time Signals

In order to compare the proposed system with the reference one in the time domain the acquired signals of each system have been recorded and shown together. Both signals have been preprocessed with a low-pass FIR filter (1 kHz cut-off frequency, 106 coefficients, and Kaiser window with 0.005 ripple) and has been aligned to match the starting time. No other processed has been applied to the signals.

**Figure 8:**
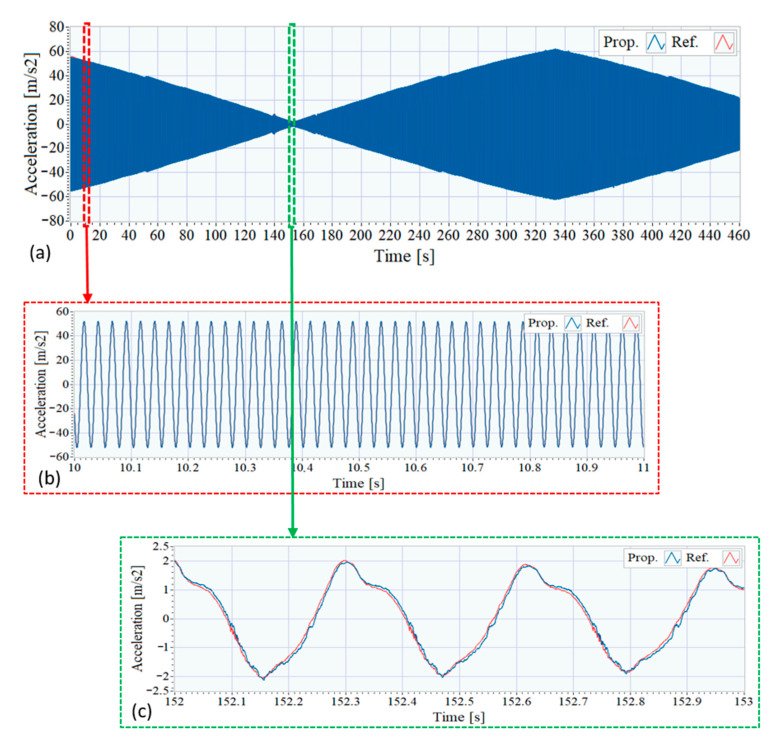
Time signals of accelerometers at the excitation sensors position. (**a**) Full signal. (**b**) Zoom between 10 and 11 s. (**c**) Zoom between 152 and 153 s.

Figure 8 shows the excitation signals registered with both systems by using the accelerometers placed on the moving mass of the shaker, in position D. Given that the shaker is excited with a constant amplitude sinusoidal sweep, the amplitude of the measured acceleration is approximately proportional to the signal frequency as can be seen in Figure 8a, which contains both captured signals: in blue, the one corresponding to the proposed system and, in red, the signal associated to the reference system. Due the similarity of these signals, two zoomed plots are shown in Figure 8b,c to emphasize their differences. As can be observed, as the input frequency decreases, and the amplitude of the signal decreases, the signals subtle differences arise mainly due to the higher noise density of the MEMS accelerometers belonging to the proposed system.

The cross-correlation between the signals acquired with the two systems has been analyzed, resulting in a correlation coefficient of 0.9998. An analysis of the SNR has been carried out to the obtained signals, and it has been found that for the excitation signals the SNR was between 50 dB and 90 dB (growing with frequency) for the proposed system, and for the reference system, the SNR was 7 dB higher, as the noise observed for the signal obtained for the reference accelerometers is 7 dB lower.

**Figure 9:**
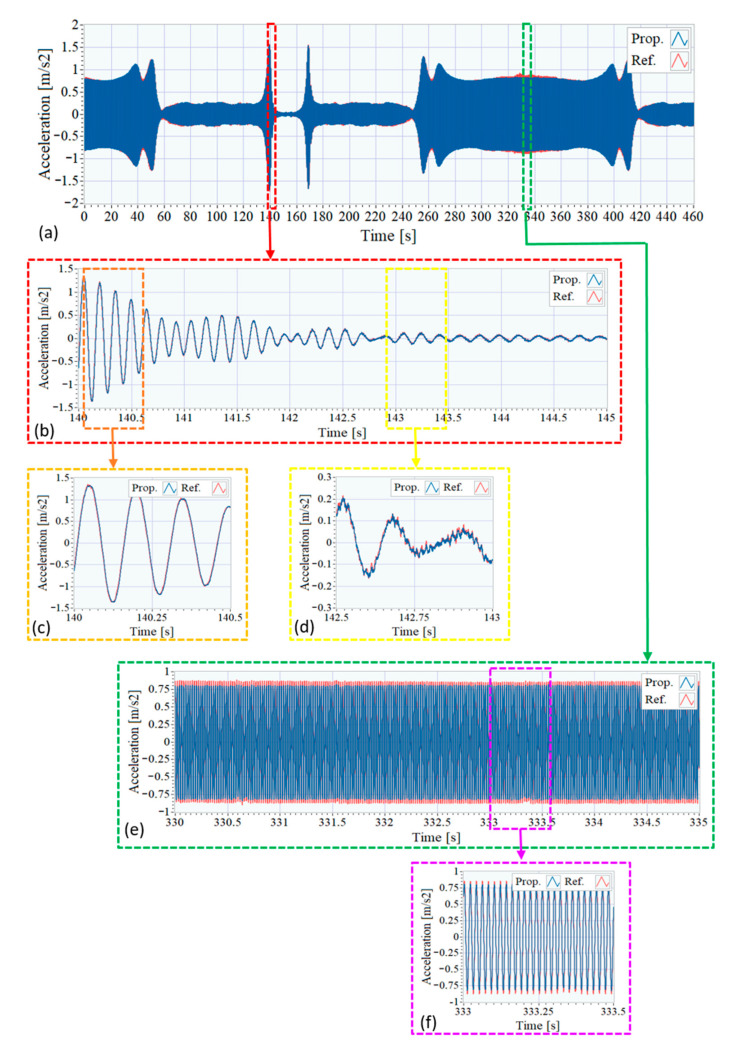
Time signals of accelerometers at position E4. (**a**) Full signal. (**b**) Zoom between 140 and 145 s. (**c**) Zoom between 140 and 140.5 s. (**d**) Zoom between 142.5 and 143 s. (**e**) Zoom between 330 and 335 s. (**f**) Zoom between 333 and 333.5 s.

In the same way, Figure 9 shows the recorded signals of the pair of accelerometers placed under the shaker, corresponding to position E4 in Figure 6, spaced only about 45 mm along the beam axis. As it is usual when the response of a structure subjected to sinusoidal excitation is obtained, high amplitudes reveal the coincidence of the input frequency with the resonance frequencies of the structure, while low amplitudes correspond to frequencies to which the structure is less sensitive.

The comparison of the measurements of these sensors again shows a great similarity in the data provided by both systems with minor variations mainly due to the more noticeable noise when the response amplitude is low, as it is shown in Figure 9d, and to their different location along the beam axis (around 325 to 345 s), as can be observed in Figure 9e,f. The small differences observed in Figure 9e,f are due to the fact that the pair of accelerometers is not exactly in the same position but about 45 mm apart in the longitudinal direction of the beam, as shown in Figure 7. This small separation does not affect the accelerations due to the main mode of bending (6.9 Hz) being compared, whose detailed zoom is shown in Figure 9. However, there may be differences in higher vibration modes (either bending or torsional). Due to the volume that the accelerometers themselves occupy; it is impossible to place them in exactly the same geometric position, and it was decided to carry out the measurements simultaneously with both systems, to ensure that the conditions under which the tests were carried out were the same.

The cross-correlation between these corresponding acquired pair of signals has been analyzed, resulting in a correlation coefficient of 0.9985. For these signals, also an analysis of the corresponding SNR has been carried out, and it has been observed that in this case, the SNR values are lower, as it is the amplitude of the signals. For these signals, the values of the obtained SNR for the proposed system were between 30 and 55 dB, which are high enough.

### 4.2. Frequency Response Functions

**Figure 10:**
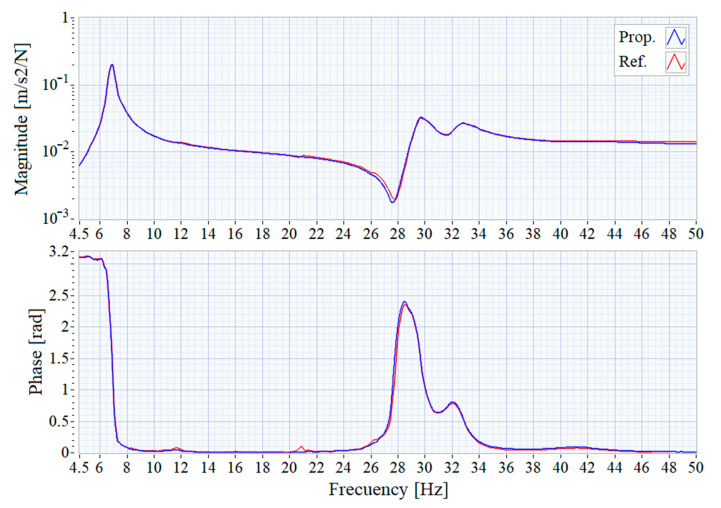
Frequency Response Function from 4.5 to 50 Hz.

The time domain signals are processed by each system to calculate the Frequency Response Functions of the test structure by using a 32,768 points (8.192 s at 4000 samples/second) Blackman window, leading to a frequency resolution of 0.366 Hz. Figure 10 shows the comparison of the results obtained by each system in the range of interest.

**Figure 11:**
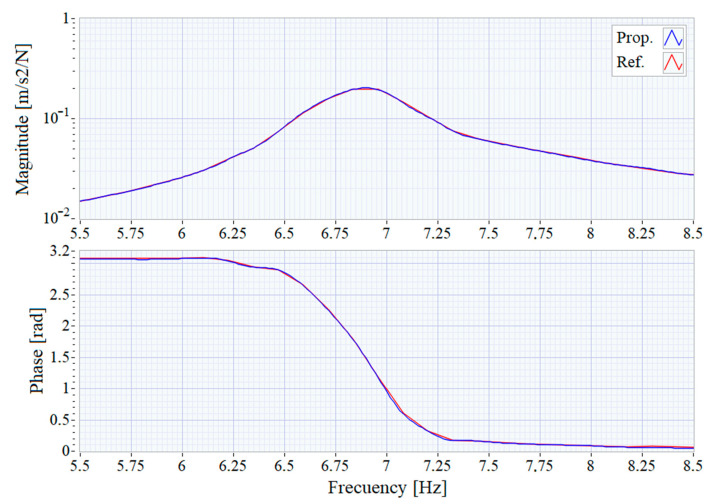
Frequency Response Function centered at the main resonance.

As can be seen, the small discrepancies observed in the time domain recordings result in minimal changes in the frequency domain responses, validating of the proposed system for modal analysis of structures. In both FRFs, a main resonance peak around 6.9 Hz can be clearly distinguished which is shown in detail in Figure 11. The FRF graphs are centered around that peak and show that the results obtained with the two systems are almost identical.

The FRF graph calculated by the proposed system presents a higher level of detail because it uses a larger DFT size (65,536) than the reference system (32,768), which would presumably lead to more precise modal analysis results. The FRF estimation algorithm implemented in the proposed system overcomes the typical limitation of the window size matching the size of the DFT, present in most FRF implementations including, among others, DewesoftX (the one used as reference system), MATLAB’s *modalfrf* function or LabVIEW’s *Estimate_FRF* block. The independence between these two parameters, the length of the window and the size of the DFT, allows improving the frequency resolution without increasing the computational burden associated to the increase of the number of DFT points. This increased resolution allows, on the one hand, to reduce the widening due to the windowing and to measure with better precision the damping factor (which is related to the sharpness of the peak) of the resonance mode. And, on the other hand, it permits to distinguish closer natural frequencies.

**Figure 12:**
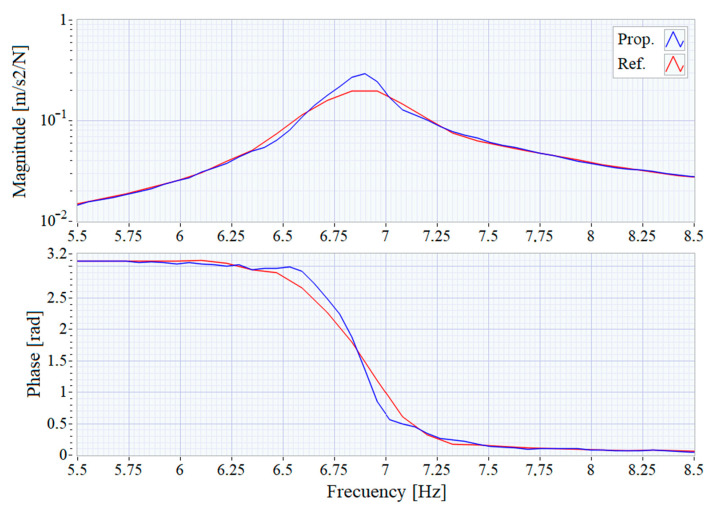
Frequency Response Function with resolution increased.

For example, Figure 12 shows the result of using a 262,144 points (65.536 s at 4000 samples/second) window to achieve a frequency resolution of 0.046 Hz. The improved resolution of FRF obtained by the proposed system shows how the amount damping of the main resonance is reduced (sharper peak) using the shorter window used by the reference system. In addition, in the FRF calculated by the proposed system, a second peak modestly appears at 7.2 Hz, while in the FRF calculated by means of the reference system, this second resonance is masked by the main resonance at 6.9 Hz, which has higher amplitude.

### 4.3. Cost

In order to quantify how much of a low-cost, the proposed system is, the cost of the equipment used as a reference system and the cost of the configuration of the proposed system used in the comparison tests has been calculated. Table 2 resumes the cost of each system without considering the computer because both systems require the use of one to work. Additionally, the cost of acquisition and processing software has not been considered because, although it is not a negligible amount in general, the proposed system uses a software developed by the authors whose price has not been estimated.

It is clear that the proposed system has a cost of 1:10 over the reference system, this ratio can be even higher when compared to brands in the upper segment of the market or if it is considered that each accelerometer of the proposed system is triaxial.

## 5. Conclusions

This paper presents a low-cost system for monitoring the structural health (SHM) based on MEMS sensors. The architecture consists of back-end units devoted to recording the signals collected by the MEMS accelerometers, as well as generating the excitation signals. The processing units are in charge of pre-processing the signals in the time domain, calculating the FRFs and, finally, performing the modal analysis of the structure. The whole process is controlled by the front-end units.

The proposed architecture based on these modules provides a scalable, reconfigurable and low-cost system compared to commercial systems based on analog sensors and acquisition systems with high-performance analog-digital converters. Therefore, it would enable the deployment of tens or even hundreds of sensors to monitor large buildings such as skyscrapers, heritage buildings such as cathedrals and churches, and in general any structure that requires massive monitoring. In this case, system synchronization could be improved for longer distances by using the TSN (Time Sensitive Networking) protocol, which is an alternative to using GPS synchronization, which would be a more expensive option.

A proven commercial acquisition system, together with IEPE analog accelerometers, has been selected to compare the performance between the two systems. Experimental testing has shown that, although the performance of analog accelerometers is better overall, both systems are equivalent for use in modal monitoring. The improved FRF calculation algorithm included allows the capture time and sampling frequency to be independent of the size of the DFT, overcoming the limitation of many modal analysis systems by achieving better frequency resolutions without increasing the computational burden.

## Figures and Tables

**Table 1:** Main characteristics of the proposed and reference system.

Characteristic	Prop. System	Ref. System
Range	±2 g, ±4 g, and ±8 g	±60 g
Digital sensitivity	3.9, 7.8 and 15.6 μg/LSB	11.9 μg/LSB
Noise density	25 µg/√Hz	3 µg/√Hz
Max. sample frequency	4 kHz	200 kHz
Bits per sample	20	24
Max. accelerometer channels	6 tri-axial	8 uni-axial

**Table 2:** Estimated cost (€) of the reference and proposed systems.

Element	Reference System			Proposed System		
	Model	Cost/Unit	Total	Model	Cost/Unit	Total
DAQ	DS-SIRIUS	6500	6500	myRIO 1900	580	580
6× Accelerometers	KS76C.100	350	2100	ADXL355 + Box	50	300
Cables	UNF to BNC	75	450	RJ45 to RJ45	8	48
Total cost			9050			928

## Data Availability

The validation data used to support the findings of this study are available from the corresponding author upon request.

## References

[1] 2020 UNESCO World Heritage List. https://whc.unescoc.org/.

[2] Farrar C.R., Worden K. (2007). An introduction to structural health monitoring. Philos. Trans. R. Soc. A.

[3] Campos J., Garcia O., Abel A., Casas J.R., Vehi J. (2009). Health Monitoring System (HMS) for structural assessment. Smart Struct. Syst..

[4] Ye X.W., Jin T., Yun C.B. (2019). A review on deep learning-based structural health monitoring of civil infrastructures. Smart Struct. Syst..

[5] Salawu O.S. (1997). Detection of structural damage through changes in frequency: A review. Eng. Struct..

[6] Farrar C.R., Doebling S.W., Nix D.A. (2001). Vibration–based structural damage identification. Philos. Trans. R. Soc. A.

[7] Mahammad A.H., Kamrul H., Ker P.J. (2018). A review on sensors and systems in structural health monitoring: Current issues and challenges. Smart Struct. Syst..

[8] Goyal D., Pabla B.S. (2016). Development of non-contact structural health monitoring system for machine tools. J. Appl. Res. Technol..

[9] Swartz R.A., Lynch J.P., Zerbst S., Sweetman B., Rolfes R. (2010). Structural monitoring of wind turbines using wireless sensor networks. Smart Struct. Syst..

[10] Gomez H.C., Fanning P.J., Feng M.Q., Lee S. (2011). Testing and long-term monitoring of a curved concrete box girder bridge. Eng. Struct..

[11] Moser P., Moaveni B. (2013). Design and development of a continuous monitoring system for the Dowling Hall Footbridge. Exp. Tech..

[12] Waidyanatha N. (2010). Towards a typology of integrated functional early warning systems. Int. J. Crit. Infrastruct. Prot..

[13] Hu C., Xiao M., Zhou H., Wen W., Yun H. (2011). Damage detection of wood beams using the differences in local modal flexibility. J. Wood Sci..

[14] Girolami A., Brunelli D., Benini L. Low-cost distributed health monitoring system for critical buildings. Proceedings of the 2017 IEEE Workshop on Environmental, Energy, and Structural Monitoring Systems (EESMS).

[15] Das S., Saha P. (2018). A review of some advanced sensors used for health diagnosis of civil engineering structures. Measurement.

[16] Ceylan H., Gopalakrishnan K., Kim S., Taylor P.C., Prokudin M., Buss A.F. (2013). Highway infrastructure health monitoring using micro-electromechanical sensors and systems (MEMS). J. Civ. Eng. Manag..

[17] Nagayama T., Sim S.H., Miyamori Y., Spencer B.F. (2007). Issues in structural health monitoring employing smart sensors. Smart Struct. Syst..

[18] Ribeiro R.R., Lameiras R.D.M. (2019). Evaluation of low-cost MEMS accelerometers for SHM: Frequency and damping identification of civil structures. Lat. Am. J. Solids Struct..

[19] Sabato A., Niezrecki C., Fortino G. (2016). Wireless MEMS-based accelerometer sensor boards for structural vibration monitoring: A review. IEEE Sens. J..

[20] Acar C., Shkel A.M. (2003). Experimental evaluation and comparative analysis of commercial variable-capacitance MEMS accelerometers. J. Micromech. Microeng..

[21] Yu Y., Ou J., Li H. (2010). Design, calibration and application of wireless sensors for structural global and local monitoring of civil infrastructures. Smart Struct. Syst..

[22] Ha D.W., Park H.S., Choi S.W., Kim Y. (2013). A Wireless MEMS-Based Inclinometer Sensor Node for Structural Health Monitoring. Sensors.

[23] Zou Y., Chen Y., Liu P. (2019). Refactoring and Optimization of Bridge Dynamic Displacement Based on Ensemble Empirical Mode Decomposition. Sensors.

[24] Zhu L., Fu Y., Chow R., Spencer B.F., Park J.W., Mechitov K. (2018). Development of a High-Sensitivity Wireless Accelerometer for Structural Health Monitoring. Sensors.

[25] Elhattab A., Uddin N., Obrien E. (2019). Extraction of Bridge Fundamental Frequencies Utilizing a Smartphone MEMS Accelerometer. Sensors.

[26] Iban N., Soria J.M., Magdaleno A., Casado C., Diaz I.M., Lorenzana A. (2018). Ad-hoc vibration monitoring system for a stress-ribbon footbridge: From design to operation. Smart Struct. Syst..

[27] Hsu T.Y., Yin R.C., Wu Y.M. (2018). Evaluating post-earthquake building safety using economical MEMS seismometers. Sensors.

[28] Iacono F.L., Navarra G., Oliva M. (2017). Structural monitoring of Himera viaduct by low-cost MEMS sensors: Characterization and preliminary results. Meccanica.

[29] Kavitha S., Daniel R.J., Sumangala K. (2016). High performance MEMS accelerometers for concrete SHM applications and comparison with COTS accelerometers. Mech. Syst. Signal. Pract..

[30] Bedon C., Bergamo E., Izzi M., Noè S. (2018). Prototyping and validation of MEMS accelerometers for structural health monitoring—The case study of the Pietratagliata cable-stayed bridge. J. Sens. Actuator Netw..

[31] Kavitha S., Daniel R.J., Sumangala K. (2016). Design and analysis of MEMS comb drive capacitive accelerometer for SHM and seismic applications. Measurement.

[32] Andò B., Baglio S., Pistorio A. A low-cost multi-sensor approach for early warning in structural monitoring of buildings and structures. Proceedings of the 2014 IEEE International instrumentation and measurement technology conference (I2MTC).

[33] Beskhyroun S., Ma Q. Low-cost accelerometers for experimental modal analysis. Proceedings of the 15th World Conference on Earthquake Engineering.

[34] Tan T.D., Anh N.T., Anh G.Q. Low-cost Structural Health Monitoring scheme using MEMS-based accelerometers. Proceedings of the 2011 Second International Conference on Intelligent Systems, Modelling and Simulation, Kuala Lumpur, Malaysia.

[35] Rosal J.E.C., Caya M.V.C. Development of Triaxial MEMS Digital Accelerometer on Structural Health Monitoring System for Midrise Structures. Proceedings of the 2018 IEEE 10th International Conference on Humanoid, Nanotechnology, Information Technology, Communication and Control, Environment and Management (HNICEM).

[36] Yick J., Biswanath M., Ghosal D. (2008). Wireless sensor network survey. Comput. Netw..

[37] Gubbi J., Buyya R., Marusic S., Palaniswami M. (2013). Internet of things (IoT): A vision, architectural elements, and future directions. Future Gener. Comput. Syst..

[38] Lamonaca F., Sciammarella P.F., Scuro C., Carnì D.L., Olivito R.S. Internet of Things for Structural Health Monitoring. Proceedings of the 2018 Workshop on Metrology for Industry 4.0 and IoT.

[39] ADXL355 3-Axis MEMS Accelerometer. https://www.analog.com/media/en/technical-documentation/data-sheets/adxl354_355.pdf.

[40] myRIO Platform. https://www.ni.com/pdf/manuals/376047c.pdf.

[41] DS-SIRIUS Data-Logger. https://dewesoft.com/products/daq-systems/sirius.

[42] KS76C.100 IEPE Accelerometers. https://mmf.de/standard_accelerometers.htm.

